# Local Anæsthesia for Incipient Alveolar Abscess

**Published:** 1869-06

**Authors:** 


### EDITORIAL DEPARTMENT.

*Local Anæsthesia for Incipient Alveolar Abscess.*—Dr. Chase in an article on "Alveolar Abscess," published in the *Missouri Dental Journal,* refers to a method pursued by Dr. Judd, of treating the incipient form of this disease by means of local anæsthesia induced by Richardson's apparatus. The following directions are given for its application:

"The tooth should be frozen its whole length, which will take from fiften to thirty minutes, *Immediate* relief from pain will ensue, and in many cases will not return."

Dr. Chase speaks of having used the spray for this purpose in one case with perfect success.

